# One health, one earth, one life: The overlooked role of veterinarians in the fight against COVID-19 and other public health emergencies in Italy

**DOI:** 10.1016/j.onehlt.2025.101207

**Published:** 2025-09-18

**Authors:** Sante Roperto, Giovanni Di Guardo

**Affiliations:** a Professor of Infectious Disease, Department of Veterinary Medicine and Animal Productions, Napoli, Via Delpino 1, 80137 Naples, Italy; b Former Professor of General Pathology and Veterinary Pathophysiology, University of Teramo, Viale Pasteur, 77, 00144 Rome, Italy

**Keywords:** Veterinary medicine, COVID19, A(H5N1) avian influenza virus, West Nile virus, One health, Zoonotic diseases, Public health communication, Italy

## Abstract

This essay explores the crucial and often overlooked role of veterinarians in managing and responding to pandemics. Despite their pivotal activities in protecting public health, veterinarians in Italy were largely absent from the media storytelling of the COVID19 pandemic, which was mostly focused on a hospital-centered perspective. Regretfully, we witness herein the occurrence of a similar situation within the media coverage in Italy of other public health emergencies, such as those caused by the highly pathogenic avian influenza A(H5N1) virus and by the West Nile virus. This article examines the asymmetry in professional representation during public health crises in Italy, thereby calling for a more inclusive approach to global health challenges, employing a One Health framework that acknowledges the connection between human, animal and environmental health. Within this framework, we also report how veterinarians contribute to food safety, zoonotic disease monitoring, epidemiological studies and ecosystem management, all of which are essential for pandemic prevention and response. Our goal in bringing these issues to light is to increase public awareness on the crucial role of veterinarians in public health risk analysis, communication and policy formulation, which will ultimately make society more resilient to potential pandemic threats in the future.

## 1 Introduction

The COVID-19 pandemic brought to light serious flaws in our worldwide health infrastructure, along with a distorted and largely asymmetric media storytelling of the veterinary profession as compared to the medical one, at least in Italy. Within such a challenging and dramatic scenario, while the Italian media correctly praised the heroic efforts of medical doctors, intensive care experts, psychologists and nurses within an almost exclusively “hospitalocentric” storytelling and perspective, veterinarians were largely if not totally ignored, despite the crucial roles played by them in the pandemic response. As a consequence, the demand for news on behalf of the general public in Italy during COVID-19 suffered a lot from this narrative distortion, which undoubtedly contributed, in turn, to feed and expand the “infodemic” dimension progressively taken by the pandemic. Noteworthy, the relevance of media on disease spread is such that its effect has been incorporated into epidemiological models [1,2]. According to the World Health Organization, approximately 70 % of emerging infectious diseases (EIDs) have either a proven or suspect origin from an animal source [3]. Nonetheless, the veterinary community, which is uniquely placed at the crossroad of animal, human and environmental health, was seldom sought for advice within the media coverage of the COVID19 pandemic, despite the consistent and significant activities performed by veterinarians in investigating and assessing cases of SARS-CoV-2 transmission from people to companion animals like cats and dogs. In this respect, although the vast majority of SARS-CoV-2 variants have developed in humans, some of them have also been “returned” by animals to people. This is clearly exemplified by a human infection case caused by a highly divergent SARS-CoV-2 lineage (B.1.641) circulating among white-tailed deer (*Odocoileus virginianus*) from the Canadian region of Ontario and sharing a quite recent common ancestry with a mink SARS-CoV-2 strain from Michigan [4]. Indeed, white-tailed deer have already been shown to be particularly susceptible to SARS-CoV-2 infection on the basis of a high homology degree of their angiotensin-converting enzyme-2 (ACE-2) viral receptor with the human one, thereby supporting in a very efficient way the intraspecies transmission of several SARS-CoV-2 variants infecting humans [5]. Naturally congregated animals like white-tailed deer are paralleled, in terms of viral susceptibility and SARS-CoV-2 re-transmission to people (from which they originally acquired the infection), by “artificially” congregated animals like farmed mink in Denmark and The Netherlands alongside pet hamsters in Hong Kong [6,7]. Despite the crucial, public health-focused, institutional activities carried out by veterinarians in the context of the COVID-19 pandemic, not even one of them was appointed as a member of the “COVID-19 Scientific Committee” that was officially established by the Italian Government to fight the COVID-19 pandemic and which was incomprehensibly dismantled after only two years since its creation [8]. Still with reference to the Italian media coverage asymmetry and distortion of the public health role played by veterinarians, it should be additionally emphasized that a similar situation is taking place, unfortunately, in the context of other ongoing public health emergencies like the avian influenza caused by the highly pathogenic avian influenza (HPAI) A(H5N1) virus alongside the West Nile flaviviral disease [9,10]. More in detail, the institutional response provided by veterinarians in tackling these alarming public health emergencies appears to be crucial in preventing the transmission of HPAI A(H5N1) virus both among susceptible domestic and wild avian and mammalian species and to people, in whom this microbial pathogen could potentially cause another catastrophic pandemic, should the virus acquire the capability to give rise to an efficient interhuman transmission chain. Furthermore, as far as concerns the lack of media coverage of veterinarians in Italy for West Nile virus (WNV) - despite the highly efficient public health responses provided by them in the fight against the virus, including anti-WNV vaccination of horses -, it should be also underlined, by paradox, that WNV is characterized by an exceedingly broad host range (including both domestic and wild mammalian and avian species alongside reptiles and even amphibians), having recently caused several cases of neuroinvasive disease in elderly or immunocompromised people with preexisting pathologies, 24 of which have also resulted in a fatal outcome [11].

The goal of this study is to address this inequality by analyzing the critical role that veterinarians play in the One Health paradigm in the event of a pandemic. It investigates how the marginalization of veterinary knowledge in the public debate may have hampered our overall knowledge of the pandemic and prevented us from developing more successful prevention plans for future outbreaks.

As a result of the COVID19 pandemic, global health systems have been permanently altered, and we are now compelled to rethink how well we are prepared for outbreaks of zoonotic diseases. We are still learning the lessons from this extraordinary catastrophe, and it is growing more evident that during crucial decision-making moments, many beneficial insights from veterinary medicine were underutilized. [12,13]

For a long time, veterinarians have led the way in monitoring and controlling diseases in animal populations. Their knowledge of infectious disease management across species boundaries gives them a rare insight into containment methods, transmission dynamics, and efficient vaccination programs. From an historical perspective, it seems interesting and appropriate, at the same time, to recall also that “Rinderpest” represented the main “historical reason” justifying the birth of the first Veterinary Medical Schools in Europe during the second half of 18th century.

Nevertheless, the media's focus on the hospital-centered narrative gave little attention to prevention at the animal-human interface, where pandemics usually start, and instead emphasized clinical treatment for human patients.

Despite being around for a while, the idea of One Health—which acknowledges the intrinsic relationship between human health, animal health, and environmental factors—has only made little headway in mainstream debates over pandemics. This was a missed opportunity to use veterinary expertise to create all-encompassing plans that tackle the underlying causes of zoonotic disease emergence, rather than just its human symptoms.

## 2 Veterinarians: Crucial but Neglected Pandemic Responders

There are several important ways that the veterinary community helps manage pandemics:

## 3 Monitoring and management of diseases that can spread from animals to humans

Veterinarians are the first line of defense against diseases that can spread from animals to humans. By routinely monitoring the numbers of domestic, livestock, and wild animals, they can spot possible disease outbreaks before they spread to human populations. They are able to identify trends in disease transmission across species barriers thanks to their training in comparative medicine, and they can also put in place control strategies that safeguard both animal and human populations [14]. Veterinarians performed important studies on SARSCoV2 susceptibility in different animal species during the COVID19 epidemic, which aided in the discovery of possible intermediate vectors and reservoir hosts. Although this knowledge was crucial for comprehending viral evolution and averting additional spillover occurrences, mainstream health communication paid little attention.

## 4 Food and animal safety

Our food supply chain is kept safe by veterinarians, who prevent the spread of diseases through animal products. Their monitoring of food processing practices, disease testing methods, and animal vaccination programs helps to prevent foodborne illnesses and regulate the transmission of zoonotic diseases. When the worldwide food system experienced pandemic-related disruptions, these contributions became even more essential because specialized veterinary input was necessary to ensure food safety while dealing with supply chain difficulties. [15]

## 5 Monitoring and epidemiological studies

Advanced epidemiological techniques have been developed by the veterinary profession to monitor and evaluate disease patterns in animal communities. These methods are immediately applicable to outbreaks of human illness, particularly those caused by zoonotic diseases. Veterinary epidemiologists provide insightful viewpoints to pandemic modeling, assisting in the prediction of disease transmission and the evaluation of intervention plans.

The experience of veterinary epidemiologists in handling infectious disease outbreaks in congregate animal environments during COVID-19 might have informed strategies for controlling spread in human congregate settings such as schools, prisons, and nursing homes. In contrast, public health messaging seldom made this link clear.

## 6 Ecosystem administration

Veterinarians help us understand how environmental changes impact disease dynamics in wildlife populations, which frequently act as reservoirs for human pathogens. Their knowledge of monitoring wildlife health aids in determining how human intrusion, climate change, and habitat disruption raise the possibility of zoonotic diseases appearing.

Veterinary insights into ecosystem health became more and more important for preventing future pandemics as COVID19 highlighted the effects of ignoring the relationship between humans, animals, and the environment. However, the mainstream discussion gave little attention to this ecological element of pandemic prevention.

## 7 Multidisciplinary cooperation in crisis response

The integration of knowledge from several fields is necessary for successful pandemic management. By collaborating with epidemiologists, virologists, ecologists, public health officials, and other doctors, veterinarians develop a holistic strategy to address health issues that cross species lines.

Because it recognizes that the health of humans, animals, and ecosystems are interdependent, the One Health approach promotes this kind of collaboration. But the COVID19 outbreak made it clear that veterinarians were underrepresented in media briefings, public health task groups, and policy talks, which restricted the possibility of genuine integrated strategies for managing the pandemic.

This exclusion is a big error since veterinarians offer distinctive viewpoints on disease management that complement medical approaches. Their experience managing disease epidemics in animal populations—which frequently require population-level interventions rather than individual treatment—provides helpful models for public health approaches during human pandemics.

## 8 The animal connection: Emerging infectious diseases

The fact that about 70 % of new infectious diseases in humans originate in animals highlights the crucial role of veterinary knowledge in pandemic prevention. West Nile virus, Ebola, influenza viruses, and SARSCoV2 are examples of zoonotic pathogens that have crossed species barriers to cause severe human disease epidemics. Due to their training, veterinarians are in a position to track these illnesses at their origin, establishing surveillance initiatives in wildlife and domestic animals in order to identify emerging pathogens before they take hold in human communities. Their knowledge of species-specific disease dynamics enables them to spot possible spillover occurrences and implement focused treatments to stop cross-species transmission.

Until after SARSCoV2 had already established global transmission chains, the early identification of wildlife markets as high-risk settings for zoonotic disease emergence, a concern long raised by veterinary and wildlife health specialists, was given insufficient attention. This constitutes a significant missed chance to take preventative measures informed by veterinary expertise.

## 9 Bridging disciplinary divides: Cultural identity and public health

Approaches to tackling public health issues, such as marine food safety and zoonotic disease management, are influenced by various professional cultures. The division between human and veterinary medicine, which is frequently thought of as distinct domains despite their evident interconnectedness, reflects underlying cultural ideas regarding the connection between human and animal health.

These professional cultural identities may impede cooperation during health crises. Despite their shared roots, the historical division of human and animal medicine in many healthcare systems has resulted in communication barriers, conflicting priorities, and occasionally competing strategies for addressing shared health concerns.

The minimal representation of veterinary viewpoints in media coverage and policy discussions during the COVID19 epidemic made these gaps especially apparent. The veterinary understanding of zoonotic disease management was given relatively little attention in comparison to hospital-based medical stories. This imbalance may have led to an excessive focus on treating the symptoms of illness in people as opposed to its roots in animals, and on treatment rather than prevention.

Bridging these cultural and disciplinary divides requires deliberate effort to incorporate diverse professional perspectives into pandemic communication and response planning. Cross-disciplinary training, integrated health systems, and inclusive policy development processes can help overcome traditional boundaries between animal and human medicine.

## 10 Models of animals used to study human diseases

An additional important contribution of veterinary medicine to human health is the significance of animal models in biomedical research. Comparative anatomy, physiology, and pathology are areas of veterinary medicine that help researchers create suitable animal models for the investigation of human diseases.

Animal models were crucial during the COVID-19 pandemic for comprehending SARS-CoV-2 pathogenesis, evaluating possible treatments, and creating vaccines. For creating these models and analyzing research results, veterinary knowledge in animal welfare, interspecies disease progression, and comparative medicine was essential.

The One Health strategy is demonstrated by this translational element of veterinary medicine, which uses knowledge across species to advance health results. However, the veterinary roots of many biomedical breakthroughs or the collaborative character of this research was seldom recognized in public discussions of COVID-19 research.

## 11 Superbugs, climate change, and interspecies disease transmission

The convergence of infectious illnesses and climate change poses new difficulties that call for veterinary competence. Extreme weather events can change animal migration patterns and upset ecosystems, which might introduce new animal reservoirs of disease to people. At the same time, shifting environmental variables have an impact on pathogen survival and vector distribution, opening up new avenues for illness transmission.

The development of superbugs that are resistant to traditional therapies is a major issue at the intersection of humans and animals. Veterinarians are essential in encouraging the responsible use of antimicrobials in animal agriculture and in keeping an eye on resistance trends in animal pathogens that might spread to people.

In mainstream media coverage, the complicated relationships between environmental change, antibiotic resistance, and the emergence of zoonotic diseases were given little attention during the COVID19 epidemic. Another aspect of veterinarians' underappreciated contribution is their expertise in monitoring these linked health risks and taking preventative actions, which were mostly lacking from public debate.

## 12 Key professional individuals are undervalued

A wider trend of devaluing specific professional skills in public health environments is reflected in the restricted exposure of veterinarians in pandemic communication. Likewise, data scientists, pathologists, and epidemiologists—all of whom are essential for comprehending the dynamics of disease and creating evidence-based therapies—did not receive much public attention during the COVID-19 response. This disparity in expert assessment can have real-world effects on how the pandemic is managed. The possible contributions of some areas of knowledge remain untapped when they are consistently ignored in public discussion and in the creation of policy. Less effective and more limited intervention strategies may result from the subsequent gaps in knowledge and viewpoint.

Deliberate steps to include a variety of expert viewpoints in health communication and policy creation are necessary to address this discrepancy. By utilizing the entire range of available experience, inclusive strategies that acknowledge the complementary character of various health disciplines may boost resilience to future health risks.

## 13 Media narrative asymmetry: Difficulties in communication

The underrepresentation of veterinary knowledge in COVID19 coverage emphasizes broader issues in health communication. The media's coverage of the pandemic often centered on frontline healthcare professionals and dramatic hospital settings, painting a picture of the response that was emotionally gripping but not comprehensive.

The public's perception of the epidemic was influenced by this narrative imbalance, which highlighted treatment above prevention and clinical response over ecological context. Because veterinarians, epidemiologists, pathologists, and other professionals whose work takes place mostly outside of hospitals are not readily visible, the general public has a fragmented understanding of how to manage pandemics.

Balanced representation of all relevant disciplines contributing to crisis response is essential for effective health communication. The inclusion of veterinary viewpoints in pandemic coverage would improve public knowledge of the dynamics of zoonotic diseases and the interdependence of health risks. By being more inclusive, this strategy might increase public participation in preventative strategies and foster support for integrated health policies.

## 14 Guidelines for inclusive health communication

We make a number of suggestions to enhance health communication in the future, based on the gaps we found in professional representation throughout the COVID19 epidemic:

1. Increase Media Expert Panels: News outlets should intentionally include veterinarians and other One Health practitioners on expert panels that address pandemic threats and zoonotic illnesses.
2. Establish comprehensive communication strategies: Public health organizations should employ communication frameworks that clearly recognize the links between human, animal, and environmental health. [16]
3. Establish Interdisciplinary Representatives: Educate health communicators to bridge disciplinary boundaries by being able to convey complicated intersectional health challenges to the general public.
4. Prioritize Prevention along with Treatment: Strike a balance between covering acute medical response and providing facts on preventative strategies at the interaction between humans, animals, and the environment.
5. Foster Narrative Diversity: Promote health coverage that reflects the whole range of experts who are involved in pandemic management, even those who are not in clinical environments.
6. Boost One Health Education: Raise awareness of the One Health approach among the general public and professionals in order to lay the groundwork for a more complete health communication strategy.
7. Establish Common Terminology: Use straightforward language to explain intricate health interrelationships that go beyond industry jargon and promotes general comprehension.

## 15 Conclusion

The COVID-19 pandemic revealed critical gaps in our approach to health communication and professional recognition during global health crises. By consistently overlooking the contributions of veterinarians and other specialists working at the human-animal-environment interface, mainstream narratives presented an incomplete picture of pandemic dynamics and missed opportunities to promote truly integrative approaches to disease management.

As we prepare for future health challenges, incorporating veterinary expertise into public health communication, policy development, and pandemic response planning will be essential. [17] The One Health framework—recognizing the fundamental interconnection between human, animal, and environmental health—offers a roadmap for this integration, emphasizing collaborative approaches that transcend traditional disciplinary boundaries.

By addressing the asymmetry in professional representation and valuing the diverse expertise needed for comprehensive health protection, we can build more resilient systems capable of preventing, detecting, and responding to emerging health threats. Veterinarians must be recognized as essential partners in this effort, bringing unique perspectives and capabilities that complement medical approaches and strengthen our collective capacity to safeguard health across species and ecosystems.

In the spirit of “One Health, One Earth, One Life,” our communication about health challenges should reflect the integrated nature of these issues, acknowledging all professionals who contribute to our understanding and management of shared health threats.

## CRediT authorship contribution statement

**Sante Roperto:** Writing – review & editing, Writing – original draft, Conceptualization. **Giovanni Di Guardo:** Writing – review & editing, Writing – original draft.

## Author statement

This manuscript is the result of a joint intellectual effort by both authors. Sante Roperto and Giovanni Di Guardo contributed equally to the conception, drafting, and critical revision of the article. Both authors approved the final version of the manuscript and agree to be accountable for all aspects of the work, ensuring its accuracy and integrity. The authors declare that there are no conflicts of interest and that no specific funding was received for this work.

## Declaration of competing interest

The authors declare that they have no known competing financial interests or personal relationships that could have appeared to influence the work reported in this short communication.

## Data Availability

No data was used for the research described in the article.
